# Genetic mapping of male sterility and pollen fertility QTLs in triticale with sterilizing *Triticum timopheevii* cytoplasm

**DOI:** 10.1007/s13353-020-00595-z

**Published:** 2020-11-23

**Authors:** Marzena Wasiak, Agnieszka Niedziela, Henryk Woś, Mirosław Pojmaj, Piotr Tomasz Bednarek

**Affiliations:** 1 Plant Breeding and Acclimatization Institute – NRI, 05-870 Radzikow, Błonie Poland; 2 Breeding Department Borowo, Plant Breeding Company-Strzelce, Czempin, Poland; 3 Breeding Department Laski, Danko Plant Breeding Company-Choryń, Laski, Poland

**Keywords:** Triticale, Genetic map, Cytoplasmic male sterility, QTL

## Abstract

Cytoplasmic male sterility (CMS) phenomenon is widely exploited in commercial hybrid seed production in economically important crop species, including rye, wheat, maize, rice, sorghum, cotton, sugar beets, and many vegetables. Although some commercial successes, little is known about QTLs responsible for the trait in case of triticale with sterilizing *Triticum timopheevii* (Tt) cytoplasm. Recombinant inbred line (RIL) F6 mapping population encompassing 182 individuals derived from the cross of individual plants representing the HT352 line and cv Borwo was employed for genetic map construction using SNP markers and identification of QTLs conferring pollen sterility in triticale with CMS Tt. The phenotypes of the F1 lines resulting from crossing of the HT352 (Tt) with HT352 (maintainer) × Borwo were determined by assessing the number of the F2 seeds per spike. A genetic map with 21 linkage groups encompasses 29,737 markers and spanned over the distance of 2549 cM. Composite (CIM) and multiple (MIM) interval mappings delivered comparable results. Single QTLs mapped to the 1A, 1B, 2A, 2R, 3B, 3R, 4B, and 5B chromosomes, whereas the 5R and 6B chromosomes shared 3 and 2 QTLs, respectively. The QTLs with the highest LOD score mapped to the 5R, 3R, 1B, and 4B chromosomes; however, the QRft-5R.3 has the highest explained variance of the trait.

**Supplementary Information:**

The online version contains supplementary material available at 10.1007/s13353-020-00595-z.

## Introduction

Triticale (*x Triticosecale* Wittmack) is a relatively young synthetic species created by hybridization of wheat and rye nearly 150 years ago (Wilson 1875), but it took 100 years until the first variety was released (Kiss 1971). The evolution of triticale as a commercial crop was slow until the mid-1980s. Since that time, the production in world area is increasing from 91 ha (1980) to 4.2 million ha (2018) (FAOSTAT 2018). The species combines grain quality and productivity typical for wheat with vigour, hardiness, disease resistance and high lysine content specific for rye (Myer and Barnett 2000). The vigorous root system and tolerance to abiotic stresses arising from rye (Niedziela et al. 2014) allow it to grow on sandy soils with low fertility. Triticale is mainly used as animal feed but holds some promise for human nutrition (Pena 2004). The development of efficient cytoplasmic male sterility system based on, i.e. CMS Tt, and introducing new triticale hybrid varieties would further stimulate the interest in breeding the species. However, many questions concerning, i.e. the genetic pool of the species, the presence of heterotic groups that could be exploited in breeding programs, development of the genetic maps dedicated for dissection of pollen sterility QTLs in triticale with CMS Tt and evaluation of markers useful for breeding programs need to be assessed.

The advances in genotyping provide an opportunity to use genomic tools in understanding the genetic structure of cultivated crops, including triticale. There were several attempts to differentiate triticale materials via DNA-based molecular markers in combination with cluster analysis (Tams et al. 2005; Kuleung et al. 2006). The analysis of the genetic diversity of 232 triticale breeding lines based on DArT markers demonstrated that the materials divided into three groups. The differences between them are not sharp, and most of the variation (86%) is due to within-population variability (Niedziela et al. 2016), suggesting that there are enough variances for the development of new varieties. Recently, advances in genotyping with DArT markers enabled genome-wide characterization of the population structure and linkage disequilibrium (LD) in a comprehensive set of 161 winter and spring triticale lines (Alheit et al. 2012). Winter and spring growth habits contributed to the population structure in triticale, and a family structure exists in both growth types. Lately, the analyses of 885 various European triticale lines revealed a lack of significant population structure but a particular grouping of materials according to their origin (Losert et al. 2017). The available information suggests that in contrast to rye (Geiger and Miedaner 2009), the distinct triticale heterotic groups may not exist or are still under development (Geiger and Miedaner 2009). Thus, the development of genetic maps based on diverse and well-characterized breeding forms that may facilitate the identification of markers towards male sterility genes are of importance.

By now, a few genetic maps of triticale are available. The first one (González et al. 2005) exploited 356 markers with an average map density of 6.9 cM and evaluated on 73 DH lines. The primary goal of the map was to study the androgenic response. The next one based on DArT, SSR, and AFLP markers in 90 doubled haploid (DH) lines was published in 2011 (Tyrka et al. 2011). Then, Alheit et al. (2011) constructed a consensus genetic map evaluation with six DH mapping populations using nearly 2000 unique DArT markers spanning over 2310 cM. The study was designed to deliver a tool for genomic approaches in triticale. Niedziela et al. (2014) constructed the map of a small fragment of the 7R chromosome encoding Al-tolerance and using association mapping suggested markers for MAS (Niedziela et al. 2015). Recently, Tyrka et al. (2015) published a map saturated with SSR, DArT, and SNP. Nevertheless, any of the maps used mapping populations useful for hybrid breeding and dissection of QTLs conferring pollen sterility trait in CMS Tt. With the development of the Next Generation Sequencing technology, genetic maps based on recombinant inbred lines (RILs) and highly saturated with molecular markers need to be created, allowing a search for markers tightly linked to putative pollen sterility genes or for association mapping and genomic selection purposes (Jannink et al. 2010; Meuwissen et al. 2001; Veyrieras et al. 2007).

Despite breeding efforts resulting in commercial triticale varieties, i.e. Hyt Prime, Hyt Max, and RGT KEAC (Geiger and Miedaner 2009), little is known on QTLs conferring pollen sterility in the CMS Tt system. There are pieces of evidence that some of them are present on the 6RL, whereas some are less effective on the 4RL chromosome (Curtis and Lukaszewski 1993). Some authors (Geiger et al. 1995; Kojima et al. 1997; Ma and Sorrells 1995) documented the importance of the sixth chromosomes of *Triticum aestivum* L., as well as the *Rf3* locus on the 1BS, in the restoration of pollen fertility. The presence of restoration genes was also evidenced on the 2A, 4B, and 6A chromosomes (Ahmed et al. 2001) and 1BS, 5AL, 5D, and 6BS using RFLPs (Ma and Sorrells 1995). In parallel to the limited information on pollen fertility restoration genes in triticale with CMS Tt (Góral 2004), there is no information on how many of them participate in male sterility. Finally, their chromosomal assignment, precise chromosomal location, not to mention putative function, is also not known (Geiger et al. 1995). All of that limit the evaluation of valuable maintainer lines in triticale. It is worth noting that published molecular studies on pollen sterility genes are usually restricted to the F2 generation (Góral 2013) as the male sterile genotypes cannot be reproduced by self-pollination. However, mapping populations on crosses between maintainer lines based on non-sterilizing cytoplasm and restorer ones (on CMS Tt) may solve the problem. Such an approach may allow evaluation of advance recombinant inbred lines (RILs). When crossed to the maternal line with sterilizing Tt cytoplasm (sterile analogue of the maintainer line), the pollen sterility phenotypes of the RILs could be assessed by analysing the number of seeds per spike of the F1 progeny (CMS-Tt). Thus, the evaluation of respective RILs required for genetic mapping purposes as well as the assessment of the phenotypic data for the quantitative trait analysis may allow for the identification of molecular markers linked to the QTLs and useful for marker-assisted selection (MAS) (Góral and Spiss 1998) purposes.

The aim of the study was to identify QTLs conferring male sterility and pollen fertility in triticale with CMS Tt using dedicated genetic map evaluated on RILs and saturated with SNP and silicoDArT markers to assess markers linked with the trait.

## Materials and methods

### Plant materials

The population of 182 recombinant inbred lines was developed in Plant Breeding Company-Strzelce (Breeding Department Borowo, Poland) by single-seed descent method from a cross between a plant from HT352 maintainer line with non-sterilizing cytoplasm (N) and cv Borwo recognized as a good restorer genotype during earlier breeding experiments. The F1 plant was bag-isolated, and the F2 seeds were ground planted. Each of the F2 plant was a starting point for the development of the RILs. The plant materials were reproduced during six consecutive seasons resulting in the F6 generation.

The HT352 maternal line with Tt cytoplasm (Tt), the analogue of the HT352 (N), was crossed by common bagging with spikes of male parents—the individual of RIL F6: (N) × Borwo. The seeds were planted on the field in two rows (10 plants per row) with a 25-cm spacing during the 2016/17 vegetation season. The HT352 (Tt) × RIL F6: (HT352 (N) × Borwo) F1 plants were bag-isolated, and the F2 seeds were collected for counting. The male sterility of maternal line was tested before crossing in two locations Borowo and Strzelce (Poland). Phenotyping was performed once in Borowo.

### Phenotyping

The number of seeds per each F1 progeny was evaluated using main spikes collected from four different plants representing 154 out of 182 RILs. Only the spikes uninfected by brown rust were considered; thus, 28 lines were omitted. The number of spikelets per spike was nearly constant (15–16 per spike). It was assumed that the F1 plants (corresponding to the respective RIL F6 line) with the highest average number of seeds per test-crossed spike was 100% fertile. Thus, the fertility of each RIL was expressed as a proportion of average seeds number per spike to the average number of seeds per spike of the most fertile line. The sterility values were calculated as the difference: 1 − fertility and transform using an arcsine square root transformation of sterility (Sokal and Rohlf 1981). The normal distribution of the trait was tested using Kolmogorov-Smirnov test with Lilliefors significance correction implemented in the XlStat software (XlStat 2019).

### DNA isolation

The total genomic DNA was extracted from ca. 100 mg of fresh leaf tissues of a single plant of each 182 RIL F6: HT352 (N) × Borwo encompassing the mapping population according to the manufacturer instruction using DNeasy Plant Mini Kit 250. DNA integrity and purity was tested via electrophoresis on 1% agarose gels stained with EtBr (0.1 μg/ml) in TBE buffer, while its quantity was measured spectrophotometrically on the NanoDrop (ND-1000).

### Genotyping

DArTseq genotyping was conducted using *Pst*I and *Taq*I digestions, and sequencing was performed with a HiSeq 2000 sequencing system (Illumina Inc., San Diego, USA) at Diversity Arrays Technology Pty Ltd., Australia (Sánchez-Sevilla et al. 2015). The resulting sequences were filtered for quality, with a cut-off at 90% confidence. The generated single nucleotide polymorphism (SNP) and silicoDArT markers were encoded to fulfil the MultiPoint Ultra-Dense software requirements established for recombinant inbred line mapping population (Ronin et al. 2015).

### Linkage map

For the construction of the genetic map, all SNP and silico DArT loci that showed no or limited deviation from the expected segregation of 1:1 (chi-squared ≤ 19.2) were employed. Moreover, markers exhibiting more than 15% of missing data were removed from the analysis. As marker phases might be assigned incorrectly, both possible phases were implemented in the MultiPoint Ultra-Dense software (Ronin et al. 2015).

Mapping consisted of the following steps: (1) Grouping of markers with zero distance and selecting a “delegate” from each group (no fewer twins than the predefined threshold were selected). (2) Except for twins of the candidates, all remaining markers were removed to the Heap. (3) Clustering the delegate markers and ordering the obtained linkage groups (LG) were performed. (4) Filling gaps and extending LG ends using markers from Heap. (5) Removal of markers violating map stability and monotonic growth of distance from a marker and its subsequent neighbours.

### Chromosomal assignment and arm orientation

The wheat LG groups were assigned to triticale chromosomes and oriented in S-L direction based on known wheat genome location of SNP and silicoDArT markers (WCM) (DArT-P/L DAT 2016), physical map of wheat (WPM) (www.wheatgenome.org 2018), the genetic map or rye (RM) (Bauer et al. 2017) and triticale (DH-T) (Tyrka et al. 2015). The synteny data were applied to verify both the S-L orientation the LGs representing rye chromosomes and the assignment of the LGs to the rye genome (Martis et al. 2013).

### Genetic map comparison

The genetic map of triticale saturated with SNP and silicoDArT markers (Tyrka et al. 2015) was used for the comparison. Similarly, the genetic map of rye (Bauer et al. 2017), the wheat consensus map V4.0 (DArT-P/L DAT 2016) and physical map of wheat (www.wheatgenome.org 2018) were applied. The order of common markers was tested by Pearson correlation in the XlStat software (XlStat 2019).

### Composite and multiple interval mapping

Composite interval mapping (CIM) and multiple interval mapping (MIM) were performed in the WinQTL Cartographer software (Silva Lda et al. 2012) following suggestions presented elsewhere. In the case of CIM, the window size was set to 1 cM and walk speed, to 1 cM, while the number of control, markers to 25. Backward regression method was applied. The significance of the QTLs was tested using 1000 permutation test.

Multiple interval mapping (MIM) was also conducted in Win QTL Cartographer (Luciano Da Costa et al. 2012). The following settings were applied in the MIM model: MIM forward search method. MIM forward regression approach was used with the following MIM selection criteria: Bayesian information criterion (BIC) → *c*(*n*) = ln(*n*); MIM search walk speed in cM: 10. The position of the QTLs was optimized, and additional QTLs were searched and epistatic effects tested. Heritability, in a narrow sense, was calculated as the additive genetic portion of the phenotypic variance available as the output from the WinQTL Cartographer.

## Results

### Phenotyping

Parental forms of the RIL F6: HT325(N) × Borwo were fully fertile. The maternal line HT352(Tt) was sterile; however, in the case of bag-isolated spikes, marginal seed setting (one to two seeds) could be sporadically present. Phenotyping of the F1 progeny of the HT352 (Tt) × RIL F6: (HT352 (N) × Borwo) showed that varying numbers of seeds were present in each of the lines (Supplementary File 1). The highest average number of seeds per spike equalled to 74. The phenotypic data concerning 28 lines infected and damaged by brown rust were assigned as missing for analysis.

The Kolmogorov-Smirnov normality tests with Lilliefors significance correction based on transformed phenotype values obtained for seeds of F1 progeny of the HT352 (Tt) × RIL F6: (HT352 (N) × Borwo) crosses indicated that pollen sterility trait showed a normal distribution (D = 0.07, *p* value (two-tailed) = 0.385 at α = 0.05). Graphical representation of the trait distribution and results of the Kolmogorov-Smirnov test are given in Supplementary File 1.

### Genetic map

**Fig. 1:**
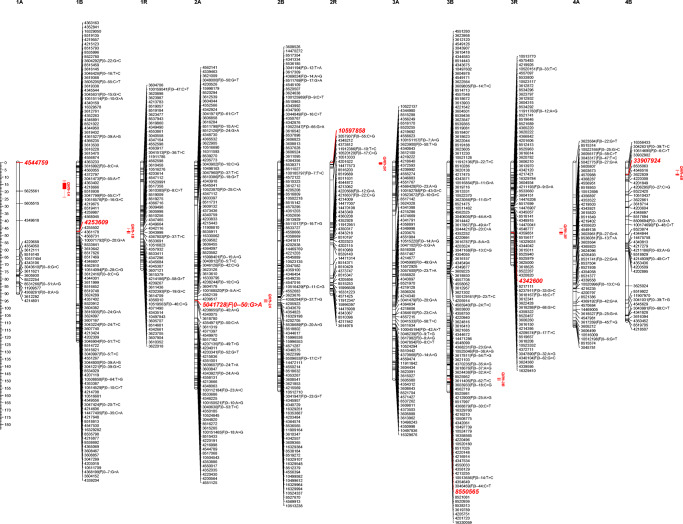

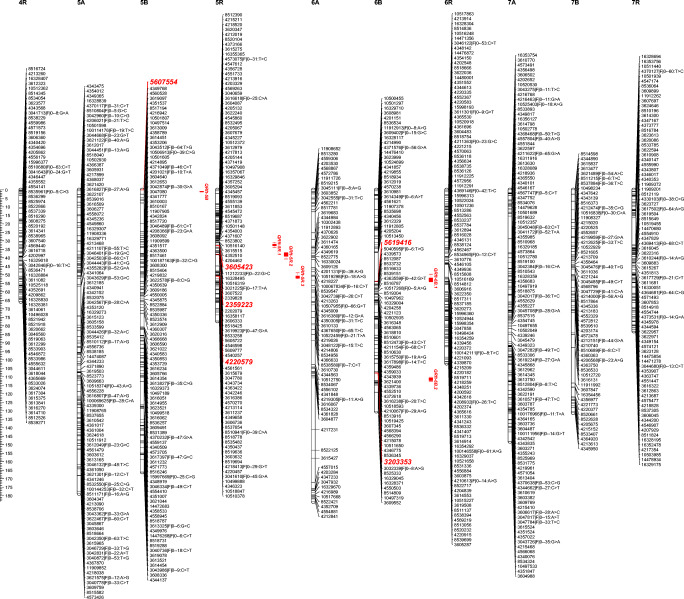
The schematic illustration of the RIL F6: HT352 (N) × Borwo genetic map. The QTLs identified by CIM are indicated by red bars (in red are the most tightly linked markers)

The RIL F6: HT352 (N) × Borwo mapping population encompassing 182 individuals genotyped with SNP and silicoDArT markers resulted in 29,671 SNP and 92,255 silicoDArT markers implemented for the construction of a genetic map. The markers with segregation distortion (chi-squared ≤ 19.2) and missing data (≥ 15% of missing data) were removed from the analysis. Assuming all markers (skeleton, redundant and added: markers with lower reliability removed during mapping steps due to segregation distortion or a large number of missing data), the map encompasses 29,737 markers. In total, 1700 skeleton and 15,568 redundant markers were mapped on 21 linkage groups (Table 1, Fig. 1). Most of the markers were silicoDArTs (23,923); the remaining were SNP (5814). The fewest number of skeleton markers mapped to 1A while the largest to 3B chromosomes. The map spanned over the distance of 2549 cM with a skeleton marker per 1.7 cM on average. The longest LG (6A) covered 183.5 cM, while the shortest (4R) 65.7 cM. Despite the high saturation of the map with molecular markers, gaps between skeleton markers were still present (Fig. 1) with the biggest spanning over 30.6 cM (6A). Eight LGs have gaps over 20 cM in length (Table 1), resulting in a lack of random distribution of markers along linkage groups (Supplementary Fig. 1). Analysis of marker density revealed that the 2R, 1A, and 6R chromosomes had the most uneven distribution, whereas the others more or less even distribution (Fig. 1).

**Table 1:** The arrangement of genetic mapping data evaluated based on 182 RIL F6: HT352 (N) × Borwo mapping population and skeleton markers (SM)

Quantifying markers and their derivatives	Chromosomes																					Characteristics			
	1A	1B	1R	2A	2B	2R	3A	3B	3R	4A	4B	4R	5A	5B	5R	6A	6B	6R	7A	7B	7R	Σ	Av/Chr	Max	Min
SM	19	109	63	99	110	47	79	117	75	50	42	73	105	102	97	70	82	109	107	61	84	1700	81	117	19
TM	112	859	1180	702	1183	645	457	745	1194	248	211	1083	548	805	1224	457	763	1677	950	930	1295	17.268	822.3	1677	112
GM	172	1323	2321	1089	1893	1457	616	1263	2655	350	332	2401	733	1185	2187	732	1117	2826	1499	1219	2367	29.737	1416	2826	172
SM/cM	4.72	1.12	1.16	1.56	1.42	1.92	1.88	1.34	0.93	2.25	2.59	0.9	1.7	1.63	0.8	2.62	1.59	0.94	1.38	2.15	0.99	35.6	1.7	4.7	0.8
TM/cM	0.8	7.02	16.19	4.54	7.56	7.15	3.08	4.74	17.08	2.21	1.94	16.48	3.06	4.85	15.85	2.49	5.86	16.43	6.41	7.09	15.56	166.4	7.9	17.1	0.8
LGL	89.6	122.4	72.9	154.7	156.4	90.2	148.6	157.1	69.9	112.4	108.6	65.7	178.8	166.1	77.2	183.5	130.3	102.1	148.1	131.2	83.2	2549	121.4	183.5	65.7
Gap	20.1	10.8	16.1	20.8	13.1	29.1	24.1	14.7	6.9	22.3	23.9	3.4	17.3	13.7	6.6	30.6	16.8	7.6	13.3	20.9	15.8	347.9	16.6	30.6	3.4

*Av/Chr* average markers per chromosome, *SM* skeleton markers, *TM* total number of mapped markers including skeleton (S) and redundant markers, *GM* includes skeleton, redundant, and added markers, *LGL* linkage group length, *SM/cM* skeleton markers per cM, *TM/cM* total number of markers per cM

The SNP and silicoDArT markers, with the known chromosomal location on wheat consensus genetic (WCM) (DArT-P/L DAT 2016), wheat physical (WPM) (www.wheatgenome.org 2018), and triticale map (DH-T) (Tyrka et al. 2015), allowed the univocal assignment of 14 LGs to the respective chromosomes. Knowing the chromosomal assignment of some markers to either short (S) or long (L) arms of the wheat genome, it was possible to orient linkage groups in the S to L direction (Supplementary Fig. 2 a, b, c). However, using DH-T (Tyrka et al. 2015), the S-L orientation of the LG representing 5B chromosome of our map was different from the one suggested by WCM and WPM. In the case of the rye genome, the marker pattern reflecting synteny was recognized (Martis et al. 2013). For example, the linkage group encompassing markers mapped to 5A, 5B, and 5D followed by those from 4A, 4B, and 4D ones were typical for 5R chromosome allowing for the proper S-L orientation of the LGs. Similar reasoning allowed the assignment of the other rye LGs. The S-L orientation was estimated based on the current marker density reflecting putative synteny or based on orientation on the other maps, including rye (RM) (Bauer et al. 2017) and DH-T (Tyrka et al. 2015) ones (Supplementary Fig. 2a). It should be stressed that the S-L orientation of the 21 linkage groups encompassing RIL F6: HT352 (N) × Borwo genetic map with WCM, WPM, RM, and DH-T (except but 5B) maps coincided. In all cases, the good collinearity of marker was observed (Supplementary Fig. 2a, b, c). The collinearity of the RIL F6: HT352 (N) × Borwo map and RM, DH-T, WCM, and WPM ones revealed no evident transpositions or translocations between the respective chromosomes (Supplementary Fig. 2a, b, c). Comparison of the correlation coefficients of the RIL F6: HT352 (N) × Borwo and WCM showed that the coefficients varied between 0.790 (5A) and 0.997 (5B) (Supplementary Table 1). In the case of the wheat physical map (WPM), the respective figures ranged from 0.686 (1A) to 0.976 (1B) for the wheat genome. A similar analysis for the rye genome, when RIL F6: HT352 (N) × Borwo was compared with the rye genetic map (RM) resulted in the range of 0.84(4R)–0.96(6R). The broadest range of correlations (0.57(2A)–1.0 (6A, 7A)) was observed when our genetic map was aligned with the triticale one. While the RIL F6: HT352 (N) × Borwo linkage groups aligned mostly in a linear way with WCM, RM, and DH-T maps, nearly in all cases, the “S” shape was observed when compared with the WPM.

### Composite and multiple interval mapping of pollen sterility

**Fig. 2:**
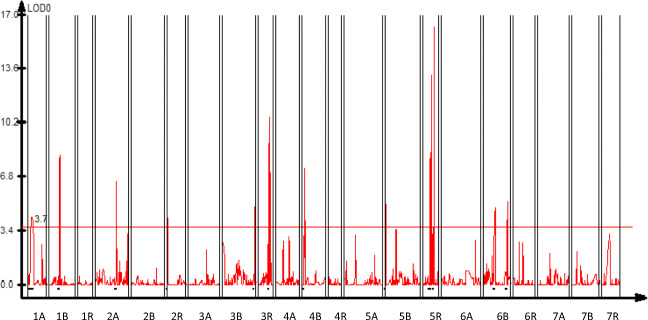
Results of composite interval mapping (CIM) of male sterility and pollen fertility in the RIL F6: HT352 (N) × Borwo. Horizontal and vertical axes represent triticale chromosomes and the log of odds (LOD) value of the tested trait, respectively. The red line indicates the level of the critical value (threshold). Black bars indicate the QTL region

Composite interval mapping showed the presence of at least 13 QTLs conferring male sterility and pollen fertility in triticale with CMS Tt identified based on the RIL F6: HT352 (N) × Borwo mapping population (Fig. 2) and shared among ten chromosomes (Table 2) with maximum log of odds (LOD) score higher than the permutation test threshold (3.7). Single QTLs were mapped to the 1A, 1B, 2A, 2R, 3B, 3R, 4B, and 5B chromosomes, whereas the 5R and 6B chromosomes have 3 and 2 QTLs, respectively. The QTLs mapped on 1A, 1B, 3B, 3R, and 4B originated from HT352 genotype, while the others from Borwo. The LOD score of the QTLs ranges from 4.23 (2R) to 16.28 (5R). The QTLs with the highest LOD score were identified on the 5R, 3R, 1B, and 4B chromosomes; however, the QRf-5R.3 has the highest explained variance of the trait (*R*^2^ = 15.1%). Out of the QTLs with the highest LOD score, four (QRft-1B, QRft-3R QRft-4B, and QRft-5R.2) have a negative additive effect. The QTLs cover from 0.5 to 11.2 cM and are bounded by tightly linked molecular markers as indicated by recombination frequency (Rec-L and Rec-R) of the LOD maximum and the nearest marker from its both sides (Table 2, Fig. 2). Markers 4253609, 10597858, 4342600, 3605423, 2359223, 4220579, and 5619416 were located exactly at the LOD maximum of the QRft-1B, QRft-2R, QRft-3R, QRft-5R.1, QRft-5R.2, QRft-5R.3, and QRft-6B.1, respectively. The most tightly linked markers are marked in red on Fig. 2.

**Table 2:** The arrangement of the composite interval mapping data of pollen sterility in triticale with CMS Tt for the RIL F6: HT352 (N) × Borwo mapping population. Rec-L and Rec-R states for the recombination frequency of the marker nearest to the QTL LOD function maximum. LOD score describes the LOD function maximum value. *R*^2^ is the proportion of variance explained by a QTL at a test site with the estimated parameters. Marker position reflects the number of the marker on the genetic map, whereas “Position” is its position in cM

No.	QTL name	QTL origin	Chromosome	Marker code	Position max LOD (cM)	LOD score	Additive	*R*^2^ (%)	Rec-L	Rec-R	Range between adjustment markers bounding QTL (from R to L)
1	QRft-1A	♀	1A	4544759	15	4.2935	− 0.0899	8.8	0.1296	0.0438	11.2
2	QRft-1B	♀	1B	4253609	47.4	8.2271	− 0.0778	6.6	0	0.0089	3.3
3	QRft-2A	♂	2A	5041728|F|0--50:G > A	94.9	6.5434	− 0.0740	5.5	0.0003	0.0056	1.5
4	QRft-2R	♂	2R	10597858	0	4.2268	0.0531	3.2	0	0.0119	1.4
5	QRft-3B	♀	3B	8550565	148.7	4.9121	0.0976	3.8	0.0003	0.0171	1.2
6	QRft-3R	♀	3R	4342600	48.2	10.5793	− 0.1439	8.8	0	0.0059	4.4
7	QRft-4B	♀	4B	33907924	8.2	7.3656	−0.1572	6.7	0.0003	0.0081	3.6
8	QRft-5B	♂	5B	5,607,554	1	5.0853	0.1387	17.8	0.0099	0.0152	0.5
9	QRft-5R.1	♂	5R	3605423	32.8	8.3856	0.1502	6.8	0	0.0094	2.9
10	QRft-5R.2	♂	5R	2359223	37.9	13.2053	− 0.2164	11.6	0	0.0033	3.8
11	QRft-5R.3	♂	5R	4220579	50.8	16.2823	0.1377	15.1	0	0.0157	3.4
12	QRft-6B.1	♂	6B	5619416	53.8	4.8811	0.0634	4.2	0	0.0034	4.5
13	QRft-6B.2	♂	6B	3203353	110.8	5.256	0.1004	11.7	0.0294	0.0137	2.5

Multiple interval mapping (Table 3) confirmed the presence of ten QTLs (except but QRft-1A, QRft-3B, and QRft-5R.1) with the same or nearly the same chromosomal positions as those detected by CIM. In all cases additive effects were observed. Two epistatic effects (QRft-3R × QRft-4B and QRft-3R × QRft-5R.3) were identified. Heritability (in a narrow sense) varied from 0.6 (QRft-5R.2) to 16% (QRft-6B.2). Additive-by-additive effects were identified for the QTLs with epistasis. In total, the heritability of all QTLs, including epistatic effects, was 68.83%.

**Table 3:** Multiple interval mapping (MIM) of pollen sterility in triticale with CMS Tt for the RIL F6: HT352 (N) × Borwo mapping population. The arrangement of the MIM. Rec-L, and Rec-R is the recombination frequency between QTL maximum and adjusted marker; *h*^2^ (%) heritability (narrow sense). The common QTLs evaluated in CIM and MIM have the same names

	Main effects							Epistatic effects							
Nr	QTL effect	Value	Chromosome	QTL name	Marker code	Rec-L	Rec-R	Chromosome	QTL name	Marker code	Rec-L	Rec-R	Position on the map (cM)	LOD	*h*^2^ (%)
1	A	− 0.0789	1B	QRft-1B	4259987	0.0001	0.01183						45.649	4.76	8.5
2	A	− 0.0567	2A	QRft-2A	5041728|F|0--50:G > A	0.0001	0.0059						94.865	2.17	1.5
3	A	− 0.0415	2R	QRft-2R	D11912386|F|0--10:T > C	0.0001	0.00767						3.352	1.34	1.4
4	A	− 0.0512	3R	QRft-3R	4342600	0.0001	0.0058						48.214	2.15	3.3
5	A	− 0.0696	4B	QRft-4B	D10514695|F|0--6:C > T	0.03006	0.00790						7.104	3.23	5.3
6	A	− 0.0692	5B	QRft-5B	4342824	0.02009	0.0040						47.831	1.81	6.9
7	A	− 0.0517	5R	QRft-5R.2	2359223	0.0001	0.0031						37.918	1.68	0.6
8	A	0.1148	5R	QRft-5R.3	4220579	0.0001	0.01590						50.805	7.44	11.5
9	A	0.0722	6B	QRft-6B.1	5619416	0.0001	0.00302						53.83	3.62	7.5
10	A	0.1161	6B	QRft-6B.2	3203353	0.03006	0.01408						110.784	2.43	16
2	AA	− 0.0489	3R	QRft-3R	D3618167|F|0--18:C > T	0.0001	0.00592	4B	QRft-4B	D10514695|F|0--6:C > T	0.03006	0.00790		1.639	3.5
2	AA	0.0382	3R	QRft-3R	D3618167|F|0--18:C > T	0.0001	0.00592	5R	QRft-5R.3	4220579	0.0001	0.01590		1.099	2.8

Based on the origin of markers conferring QTL regions, five QTLs originated from the maternal line, whereas the others were due to the parental form (Table 2). Four QTLs explained more than 0.1 of the phenotypic variance of pollen fertility restoration. Male sterility QTLs were assigned to wheat genome 1A, 1B, 3B, and 4B chromosomes, except on one localized on 3R. Male sterility QTLs explained less than 0.1 of phenotypic variance each.

CIM and MIM analysis identified ten common QTLs, namely, QRft-1B, QRft-2A, QRft-2R, QRft-3R, QRft-4B, QRft-5B, QRft-5R.2, QRft-5R.3, QRft-6B.1, QRft-6B.2, QRft-3R, and QRft-3R. Both methods indicated exactly the same chromosome positions for six QTLs located on 2A, 3R, 5R, and 6B chromosomes.

## Discussion

The RIL F6: HT352 (N) × Borwo map presented in the given study is in concordance with either wheat consensus (DArT-P/L DAT 2016), physical (www.wheatgenome.org 2018), rye (Bauer et al. 2017), and triticale genetic (Tyrka et al. 2015) maps. However, some discrepancies concerning linkage group length, the extent of gaps, or number of markers encompassing the map even if maps of the same species based on the same marker types were considered are still present.

Our mapping procedure assumed the selection of markers with limited segregation distortion (chi-squared ≤ 19.2) and elimination of markers with missing data (more than 15% of missing data). The restrictive limitations on markers used for the construction of our genetic map resulted in exclusion from mapping procedures of nearly 75% of markers forming initial set (initial set. 121,926 markers; selected for mapping, 29,737). The successive mapping steps reduced the figure to 17,268 (1700 skeleton) markers (ca. 14% of the initial set) what might have reduced the overall map length. The shorter length of the RIL F6: HT352 (N) × Borwo genetic map (2649 cM) than the one based on DH-T lines (4910 cM) (Tyrka et al. 2015) might be the result of many recombination events (ca. 80 based on skeleton markers) and the restricted number of chromosomal changes not so frequent during generative propagation and rigorous marker selection assay. It is not without significance that our mapping population is based on 182 individuals in contrast to 92 used in the case of the DH-T genetic map (Tyrka et al. 2015), which may impact on map resolution and its size. Alternatively, the reduced size of the RIL F6 genetic map in contrast to the DH-T one (Tyrka et al. 2015) might be the result of mapping procedure implemented in the MultiPoint Ultra-Dense software which prefers insertion of markers between adjustment markers, leading to the reduction of genetic map distances.

Furthermore, the reduced size of the wheat and rye genomes of the RIL F6: HT352 (N) × Borwo mapping population was observed, due to the lack of some chromosomal parts of some rye (1R, 2R, and 7R) and wheat (2A, 3A, 6A, 7A, 4B, and 6B) genomes, compared with the maps of respective species used in the study. The observed reduction could be evidenced based on Durum wheat consensus map (Maccaferri et al. 2015) covering over 2631 cM or wheat consensus map covering 2173.82 cM (DArT-P/L DAT 2016), whereas all linkage groups representing the wheat genome of the RIL F6: HT352 (N) × Borwo genetic map of triticale cover 1987.8 cM. In the case of the rye genome, the genetic map of the RIL F6: HT352 (N) × Borwo population covers 561.2 cM, whereas the consensus map of rye (Milczarski et al. 2011), 1593 cM. The observed phenomenon may reflect genome changes during the establishment of triticale (Wilson 1875). Remarkably, the reduction of the rye genome size in triticale was evidenced by Tyrka et al. (2015). The rye genome equalled to 1084.5 cM (the 7R chromosome was missing), coinciding with the data demonstrating that some rye chromosomes in triticale showed an apparent reduction in the size of C-bands at one or more telomeres, compared with normal rye (Seal and Bennet 2011). Thus, the shorter gaps and the elimination of some genomic regions in triticale due to, i.e. the formation of a new species, may also explain why the rye genome of the RIL F6: HT352 (N) × Borwo population was shorter than in the case of respective rye (Bolibok-Brągoszewska et al. 2009).

One of the aspects of our study was the comparative analysis of the RIL F6: HT352 (N) × Borwo genetic map with the consensus (DArT-P/L DAT 2016; Marone et al. 2012) and physical map of wheat (www.wheatgenome.org 2018), the genetic map of rye (Bauer et al. 2017; Milczarski et al. 2016), and the triticale (Tyrka et al. 2015). The consensus map of wheat (DArT-P/L DAT 2016) (DArT-P/L DAT 2016) and the map of triticale (Tyrka et al. 2015) resulted in the sufficient number of shared markers allowing linkage group orientation assessment based on the known location of SNP and silicoDArT markers on short (S) and long (L) arms of wheat genome as well as the S-L orientation of rye chromosomes. Comparison of wheat consensus (DArT-P/L DAT 2016), as well as physical (www.wheatgenome.org 2018) maps and our triticale (Tyrka et al. 2015) one, showed that the respective wheat chromosomes were most highly correlated. The only exception was the 5B linkage group as it exhibited a negative correlation with the respective LG on DH-T triticale map (Tyrka et al. 2015). As we have observed, the positive marker order correlation of our linkage group with the 5B chromosomes of wheat consensus (DArT-P/L DAT 2016) and physical (www.wheatgenome.org 2018) map and the S-L orientation of the 5B group remained unchanged. Similarly, the RIL F6: HT352 (N) × Borwo–based triticale rye genome map correlated well with rye (Bauer et al. 2017) and DH-T (Tyrka et al. 2015) maps.

At last, the RIL F6: HT352 (N) × Borwo genetic map of triticale is well saturated with molecular markers of low heterozygosity, indicating that the lines are quite uniform. Assuming that 10 cM marker density (in our case, it is 1.7 cM) allows an accurate estimation of QTL positions in the case of a population size between 100 and 200 (Li et al. 2010), the RIL F6: HT352 (N) × Borwo genetic map based on SNP and silicoDArT markers distributed among 21 linkage groups assigned to triticale chromosomes fulfils the requirements for QTL analysis.

By now, most of the materials used for molecular studies of pollen fertility restoration QTLs in rye (Stojałowski et al. 2004) and triticale (Stojałowski et al. 2013) are based on the F2 progeny of biparental mapping populations (Miedaner et al. 2000; Stojałowski et al. 2004). Such an approach limits the number of putative crossing-overs leading to the necessity of using large mapping populations if a high resolution of genetic maps is required (Stojałowski et al. 2013). To avoid genotyping expenses and to accumulate recombination events, recombinant inbred lines could be exploited (Stojałowski et al. 2011). In our study, the F6 progeny of the biparental mapping population based on maintainer on non-sterilizing (N) cytoplasm and restorer line on CMS Tt was utilized. As the F1 plants of the RIL population HT352(N) × Borwo were on normal cytoplasm, further generations were easily evaluated via SSD approach without loss of QTLs responsible for male sterility and pollen fertility trait in triticale with CMS Tt. Although RILs are often used in studies on quantitative traits, to our best knowledge, in the case of CMS Tt, they were employed for the first time.

The evaluation of male sterility and pollen fertility trait via visual pollen fertility scale proposed by Geiger and Morgenstern (1975) or Góral (2002) seems to be hardly possible in triticale as plant materials based on CMS Tt are easy for restoration, and the visual scale is often binary. The alternative option is to score the number of seeds per spike. Such an approach is also supported by the fact that in contrast to rye, triticale is self-compatible, reducing the possibility of overestimation of sterile plants. It should be noted that the number of seeds per spike may depend on the number of spikelets. However, this is not the case in our study as the number of spikelets varied from 15 to 16, and the normalization for spikelets was omitted. Finally, we observed that male sterility of maternal line (HT352 cms Tt) was preserved with minor fluctuations in different environments (from time to time, two to three seeds were observed in the case of some spikes of plants) which is in agreement with results presented by Góral et al. (2006). All phenotypic data were evaluated from a single environment without repetition in successive years but is noticeable that the variation within most hybrid combinations is moderate, which suggests that results are trustworthy. Nevertheless, the interpretation of the data in terms of QTL analysis must be treated with caution.

It should be stressed that under bag-isolators, brown rust may infect spikes, reducing or blocking seed setting. Special care needs to be taken to avoid or minimize the extent of such infections. In the case of the F1 progeny of the HT352 (Tt) × (RIL F6: HT352 (N) × Borwo), some spikes were severely damaged by brown rust, resulting in lack of or small degenerated seeds with a wrinkled surface. Seeds of such spikes or infected spikes with missing seeds were treated as missing data. To have phenotypic data corresponding to as many as possible RIL F6: HT352 (N) × Borwo lines, the data from moderately and partly infected materials with normal seeds were scored. Thus, due to brown rust infection, we have lost about 15% of phenotypic data which should not be a problem assuming that our mapping population consisted of 182 RILs.

In order to make our results comparable with other data, we have performed normalization expressing male sterility relative to the number of seeds of the most successful line. Furthermore, raw data were Bliss-transformed to fulfil requirements of normal distribution of the trait (Broman 2001). The transformed phenotypic data followed normal distribution based on Kolmogorov-Smirnov test (Supplementary File 1).

By now, the QTLs responsible for pollen fertility restoration in triticale with CMS Tt were mapped to 6RL and 4RL chromosomes (Curtis and Lukaszewski 1993). The importance of the 6R chromosome was suggested by Stojałowski (Stojałowski et al. 2013). The same authors indicated that the trait is covered by the remaining chromosomes of the sixth homology group (6A and 6B). It is noteworthy, that we did not identify a QTL for 6R that is supposed to be a good restorer for the Tt cytoplasm. Most probably, this QTL was eliminated during breeding programs since we did not identify it using distinct parental lines in other currently running projects. The other putative QTLs were also suggested on the 1B and 3A (3B) chromosomes (Stojałowski et al. 2013). Interestingly, the presence of effective restorer gene on 1BS (as well as those on 2A and 4B) described in wheat with CMS Tt (Ahmed et al. 2001; Kojima et al. 1997; Ma and Sorrells 1995) was not univocally confirmed in triticale (Stojałowski et al. 2013). In our materials, the 1B QTL confers male sterility rather than pollen fertility and was detected only by CIM. The lack of the 1BS pollen fertility QTL might be the effect of parental forms that missed the QTL, or it was eliminated during selection programs. Not surprisingly, our results comprising composite and multiple interval mapping demonstrated that the trait is determined by numerous QTLs located on both wheat and rye chromosomes. Moreover, one cannot exclude that some of the *Rf* genes may not restore fertility on their own and that at least some of the QTLs on subgenomes A and B are modifiers as suggested in common wheat (Würschum et al. 2017). All of the QTLs explain limited percentages of phenotypic variance with the highest values of the LOD function for the QRft-5R.2 (13.2) and QRft-5R.3 (16.2) ones. Interestingly, the QRft-5R.3 (identified both by CIM and MIM), in contrast to the QRft-5.2 (CIM and MIM), had a relatively high value of the heritability (*h*^2^, 11.5 vs 0.6). Moreover, the QRft-5R.3 covers only 3.4 cM and has tightly linked markers, which may suggest this chromosome region as a putative QTL for MAS purposes. Interestingly, our analysis showed that the trait is expressed by some QTLs located on rye genome not identified earlier (Curtis and Lukaszewski 1993), namely, those on the 2R, 3R, and 5R chromosomes. On the other hand, our results are congruent with previous studies (Ahmed et al. 2001; Curtis and Lukaszewski 1993; Kojima et al. 1997; Ma and Sorrells 1995; Stojałowski et al. 2013), indicating the multigenic nature of male sterility and pollen fertility trait in triticale with CMS Tt. They also support the notion that individual QTLs conferring the trait explain a small fraction of phenotypic variance (Stojałowski et al. 2013). However, this may not always be the case as the QRft-5B and QRft-5R.3 explained ca 15.1 and 17.8 of variance based on *R*^2^ values, respectively. It should be stressed that our study confirmed the role of the QTLs located on the sixth homology group as two of them (QRft-6B.1 and QRft-6B.2) were identified. The QRft-6B.2 was the one with the highest heritability value (*h*^2^ = 16) but with low level of the LOD score (4.8) as indicated by MIM and CIM, respectively. Both QTLs covered a small genome. Multiple interval mapping method has also demonstrated the presence of epistasis between the QRft-3R and QRft-4B and the QRft-3R and QRft-5R.3, but the effects were weak. QTL analyses are congruent with phenotypic data indicating multigenic nature of a trait.

In the given study, the RIL F6: HT352 (N) × Borwo–based genetic map dedicated to the identification of pollen sterility QTLs in the CMS Tt system of triticale was constructed. The map is saturated with numerous SNP and silicoDArT markers, and the evaluated linkage groups have acceptable linkage disequilibrium values, which maps the map useful for the QTL mapping purposes. A set of male sterility and pollen fertility QTLs was evaluated by CIM and MIM procedures indicating the prevailing role of the 5R (QRft-5R.2) and 6B (QRft-6.1) chromosomes in the trait expression in triticale with CMS Tt. Moreover, some of the QTLs might be considered as the candidates for the MAS if SNP markers are converted to the specific PCR conditions and work on a wide range of materials. Our study is the first one to demonstrate not only that the trait may be expressed by numerous QTLs explaining a small fraction of the phenotypic variance of the trait but also that some QTLs might be relatively robust. We cannot exclude, however, that the identification of such QTLs is the kind of bias reflecting parental effect. Thus, this hypothesis needs to be tested on other materials.

## Supplementary information

ESM 1 (DOCX 98 kb)

ESM 2 (DOCX 213 kb)

ESM 3 (DOCX 271 kb)

ESM 4 (DOCX 289 kb)

ESM 5 (DOCX 88 kb)

ESM 6 (DOCX 24 kb)

ESM 7 (XLSX 1296 kb)
