# Supplementary material for: Genetic mapping of male sterility and pollen fertility QTLs in triticale with sterilizing Triticum timopheevii cytoplasm
Source: J Appl Genet. 2020 Nov 23;62(1):59–71. doi: 10.1007/s13353-020-00595-z (PMC7822802; doi:10.1007/s13353-020-00595-z)
Supplement: Supplementary file 1 — (DOCX 98 kb) [file 13353_2020_595_MOESM1_ESM.docx]

**Supplementary figure 1.** Marker distribution along linkage groups representing the RIL F6: HT352 (N) x Borwo mapping population chromosomes. The skeleton, redundant and added markers are indicated in blue whereas skeleton and redundant markers in yellow. Number or respective markers is indicated on the axis of ordinates whereas the length of each linkage group in cM on the axis of abscissae.

|  |  |  |
| --- | --- | --- |
|  |  |  |
|  |  |  |
|  |  |  |
|  |  |  |
|  |  |  |
|  |  |  |
