# Supplementary material for: Genetic mapping of male sterility and pollen fertility QTLs in triticale with sterilizing Triticum timopheevii cytoplasm
Source: J Appl Genet. 2020 Nov 23;62(1):59–71. doi: 10.1007/s13353-020-00595-z (PMC7822802; doi:10.1007/s13353-020-00595-z)
Supplement: Supplementary file 2 — (DOCX 213 kb) [file 13353_2020_595_MOESM2_ESM.docx]

**Supplementary figure 2a.** Graphical illustration of the triticale rye chromosome collinearity of the RIL F6: HT352 (N) x Borwo with the a triticale (DH-T) and rye (RM) maps. Red, yellow, and blue dots represent skeleton, redundant and added markers.

|  | **RIL F6: HT352 x Borwo vs RM and DH-T maps** | |
| --- | --- | --- |
| **Chrom.** | **RM** | **DH-T** |
| **1R** |  |  |
| **2R** |  |  |
| **3R** |  |  |
| **4R** |  |  |
| **5R** |  |  |
| **6R** |  |  |
| **7R** |  | 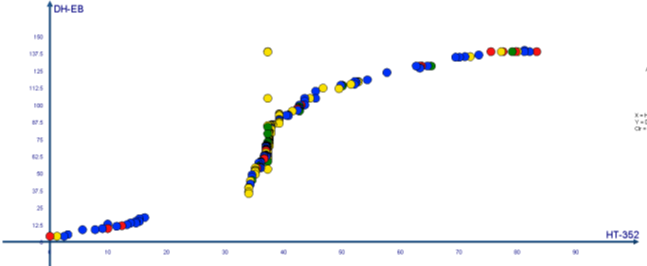 |
