# Supplementary material for: Genetic mapping of male sterility and pollen fertility QTLs in triticale with sterilizing Triticum timopheevii cytoplasm
Source: J Appl Genet. 2020 Nov 23;62(1):59–71. doi: 10.1007/s13353-020-00595-z (PMC7822802; doi:10.1007/s13353-020-00595-z)
Supplement: Supplementary file 3 — (DOCX 271 kb) [file 13353_2020_595_MOESM3_ESM.docx]

**Supplementary figure 2b**. Graphical illustration of the triticale wheat “A” genome chromosome collinearity of the RIL F6: HT352 (N) x Borwo with the wheat consensus (WCM), wheat physical (WPM) and triticale (DH-T) maps. Red, yellow, and blue dots represent skeleton, redundant and added markers.

| **Chrom.** | **RIL F6: HT352 x Borwo vs. WCM, WPM and DH-T maps** | | |
| --- | --- | --- | --- |
|  | **WCM** | **WPM** | **DH-T** |
| **1A** |  |  |  |
| **2A** |  |  |  |
| **3A** |  |  |  |
| **4A** |  |  |  |
| **5A** |  |  |  |
| **6A** |  |  |  |
| **7A** |  |  |  |
