# Supplementary material for: Genetic mapping of male sterility and pollen fertility QTLs in triticale with sterilizing Triticum timopheevii cytoplasm
Source: J Appl Genet. 2020 Nov 23;62(1):59–71. doi: 10.1007/s13353-020-00595-z (PMC7822802; doi:10.1007/s13353-020-00595-z)
Supplement: Supplementary file 5 — (DOCX 88 kb) [file 13353_2020_595_MOESM5_ESM.docx]

**Supplementary figure 3.** Frequency and accumulative frequency of marker density. Distribution of skeleton marker density is displayed as a histogram and an accumulative distribution.

**
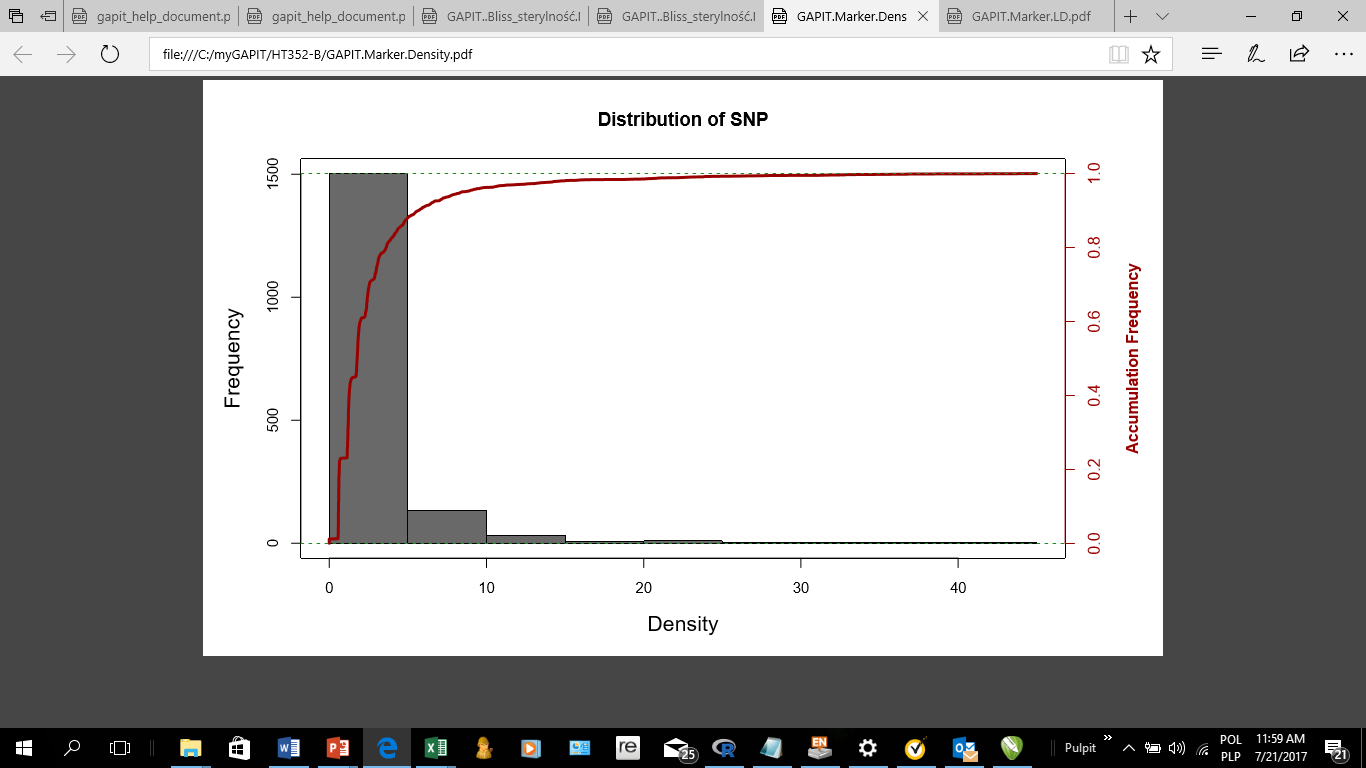
**
