# Supplementary material for: Genetic mapping of male sterility and pollen fertility QTLs in triticale with sterilizing Triticum timopheevii cytoplasm
Source: J Appl Genet. 2020 Nov 23;62(1):59–71. doi: 10.1007/s13353-020-00595-z (PMC7822802; doi:10.1007/s13353-020-00595-z)
Supplement: Supplementary file 6 — (DOCX 24 kb) [file 13353_2020_595_MOESM6_ESM.docx]

**Supplementary table 1.** The arrangement of statistics illustrating the correlation of the RIL F6: HT352 (N) x Borwo with triticale (DH-T), wheat consensus map (WCM) V4, wheat physical (WPM) and rye genetic (RM) maps (r -correlation coefficient, p- probability of obtaining test results, r2 - coefficient of determination).

|  | **RIL F6: HT352 (N) x Borwo vs MAPS** | | | | | | | | | | | |
| --- | --- | --- | --- | --- | --- | --- | --- | --- | --- | --- | --- | --- |
|  | **DH-T** | | | **WCM** | | | **WPM** | | | **RM** | | |
| **Chromosome**  **Statistics** | ***r*** | ***p*** | ***r^2^*** | ***r*** | ***p*** | ***r^2^*** | ***r*** | ***p*** | ***r^2^*** | ***r*** | ***p*** | ***r^2^*** |
| **1A** | 0.573 | 0.008 | 0.329 | 0.808 | 0.0001 | 0.652 | 0.686 | 0.0001 | 0.471 | - | - | - |
| **1B** | 0.979 | 0.0001 | 0.958 | 0.803 | 0.0001 | 0.645 | 0.976 | 0.0001 | 0.953 | - | - | - |
| **1R** | 0.887 | 0.0001 | 0.788 | - | - | - | - | - | - | 0.891 | 0.0001 | 0.794 |
| **2A** | 0.570 | 0.0001 | 0.325 | 0.977 | 0.0001 | 0.954 | 0.841 | 0.0001 | 0.708 | - | - | - |
| **2B** | 0.885 | 0.0001 | 0.784 | 0.967 | 0.0001 | 0.935 | 0.881 | 0.0001 | 0.776 | - | - | - |
| **2R** | 0.939 | 0.0001 | 0.882 | - | - | - | - | - | - | 0.940 | 0.0001 | 0.883 |
| **3A** | 0.961 | 0.0001 | 0.924 | 0.975 | 0.0001 | 0.951 | 0.947 | 0.0001 | 0.896 | - | - | - |
| **3B** | 0.856 | 0.0001 | 0.733 | 0.970 | 0.0001 | 0.942 | 0.894 | 0.0001 | 0.799 | - | - | - |
| **3R** | 0.937 | 0.0001 | 0.878 | - | - | - | - | - | - | 0.954 | 0.0001 | 0.911 |
| **4A** | 0.825 | 0.0001 | 0.681 | 0.924 | 0.0001 | 0.854 | 0.697 | 0.0001 | 0.485 | - | - | - |
| **4B** | 0.918 | 0.0001 | 0.843 | 0.975 | 0.0001 | 0.951 | 0.780 | 0.0001 | 0.608 | - | - | - |
| **4R** | 0.831 | 0.0001 | 0.691 | - | - | - | - | - | - | 0.840 | 0.0001 | 0.706 |
| **5A** | 0.941 | 0.0001 | 0.885 | 0.790 | 0.0001 | 0.624 | 0.903 | 0.0001 | 0.815 | - | - | - |
| **5B** | -0.957 | 0.0001 | 0.916 | 0.997 | 0.0001 | 0.994 | 0.834 | 0.0001 | 0.696 | - | - | - |
| **5R** | 0.929 | 0.0001 | 0.862 | - | - | - | - | - | - | 0.960 | 0.0001 | 0.922 |
| **6A** | 1.000 | 0.0001 | 1.000 | 0.968 | 0.0001 | 0.938 | 0.704 | 0.0001 | 0.496 | - | - | - |
| **6B** | 0.917 | 0.0001 | 0.842 | 0.921 | 0.0001 | 0.849 | 0.852 | 0.0001 | 0.726 | - | - | - |
| **6R** | 0.903 | 0.0001 | 0.815 | - | - | - | - | - | - | 0.960 | 0.0001 | 0.922 |
| **7A** | 1.000 | 0.0001 | 1.000 | 0.979 | 0.0001 | 0.958 | 0.864 | 0.0001 | 0.747 | - | - | - |
| **7B** | 0.660 | 0.0001 | 0.436 | 0.986 | 0.0001 | 0.973 | 0.745 | 0.0001 | 0.555 | - | - | - |
| **7R** | 0.908 | 0.0001 | 0.825 | - | - | - | - | - | - | 0.932 | 0.0001 | 0.868 |
